# An approach to uncover the relationship between 17b-estradiol and ESR1/ESR2 ratio in the regulation of canine corpus luteum

**DOI:** 10.3389/fvets.2022.885257

**Published:** 2022-08-02

**Authors:** Antenor Pereira Bonfim Neto, Ana Paula Mattoso Miskulin Cardoso, Renata dos Santos Silva, Liza Margareth Medeiros de Carvalho Sousa, Ines Cristina Giometti, Mario Binelli, Stefan Bauersachs, Mariusz Pawel Kowalewski, Paula de Carvalho Papa

**Affiliations:** ^1^School of Veterinary Medicine and Animal Science, University of São Paulo, São Paulo, Brazil; ^2^Institute of Health Sciences, Paulista University, São Paulo, Brazil; ^3^Faculty of Veterinary Medicine, University of Western São Paulo, Presidente Prudente, Brazil; ^4^Department of Animal Sciences, University of Florida, Gainesville, FL, United States; ^5^Institute of Veterinary Anatomy, Vetsuisse Faculty, University of Zurich, Zurich, Switzerland

**Keywords:** dog, diestrus, *ESR1*, *ESR2*, 17b-estradiol, progesterone

## Abstract

The canine corpus luteum (CL) is able to synthetise, activate and deactivate 17b-estradiol (E2) and also expresses nuclear estrogen receptors in a time-dependent manner during diestrus. Nevertheless, we are still missing a better comprehension of E2 functions in the canine CL, especially regarding the specific roles of estrogen receptor alpha (ERa) and ERb, encoded by *ESR1* and *2*, respectively. For that purpose, we analyzed transcriptomic data of canine non-pregnant CL collected on days 10, 20, 30, 40, 50 and 60 of diestrus and searched for differentially expressed genes (DEG) containing predicted transcription factor binding sites (TFBS) for *ESR1* or *ESR2*. Based on biological functions of DEG presenting TFBS, expression of select transcripts and corresponding proteins was assessed. Additionally, luteal cells were collected across specific time points during diestrus and specificity of E2 responses was tested using ERa and/or ERb inhibitors. Bioinformatic analyses revealed 517 DEGs containing TFBS, from which 67 for both receptors. In general, abundance of predicted *ESR1* targets was greater in the beginning, while abundance of *ESR2* targets was greater in the end of diestrus. *ESR1*/*ESR2* ratio shifted from an increasing to a decreasing pattern from day 30 to 40 post ovulation. Specific receptor inhibition suggested an ERa-mediated positive regulation of CL function at the beginning of diestrus and an ERb-mediated effect contributing to luteal regression. In conclusion, our data points toward a broad spectrum of action of E2 and its nuclear receptors, which can also act as transcription factors for other genes regulating canine CL function.

## Introduction

The canine corpus luteum (CL) has been considered as a source and target of steroid hormones, mainly progesterone (P4) and 17b-estradiol [E2, (1)]. The local production and role of P4 has been addressed earlier and there is a consensus that P4 is the primary hormone that maintains corpus luteum function and consequently, pregnancy (2, 3). Regarding E2, it is known that the canine CL produces E2 (4, 5), expresses enzymes for E2 activation (STS, steroid sulfatase) and deactivation [SULT1E1, sulphotransferase 1E member 1 (6)], and that the expression of E2 receptors (ERa and ERb, encoded by *ESR1* and *ESR2* genes, respectively) fluctuates during diestrus in pregnant and non-pregnant CL (1, 6). The ratio between *ESR2*/*ESR1* varies along non-pregnant diestrus, being greater on day 40 post-ovulation (p.o.) compared to days 10, 20, 30 and 60 p.o. (6). The gene *HSD17B7*, encoding 17b-hydroxylase, the enzyme converting estrone to E2, increases from day 10 to 20 and further to day 40 p.o. (6), whereas *CYP19A1*, encoding P450 aromatase, is greater expressed between days 35 and 45 p.o. (1). Expression of *STS* mRNA peaks on day 30 p.o., that of *SULT1E1*, on days 50 and 60 p.o. (6), indicating an established local machinery modulating E2 production and activity, which shows a turning point around day 40 p.o., when early luteal regression starts.

The effect of E2 on luteal function appears to be species-specific. For example, E2 presents a luteotropic role, as observed in rabbits and rats or a luteolytic function, as observed in humans and cattle (7–9). The luteotropic or luteolytic effect depends apparently on which receptor E2 binds to, ERa or ERb, which belong to the nuclear receptor family of intracellular receptors, exhibiting similar structures, but distinct regulatory functions. In general, ERa promotes cell proliferation, whereas ERb appears to have an anti-proliferation role (10). Moreover, in cells that express both receptors, it appears that ERb inhibits the transcriptional activity of ERa (11); consequently, E2 signaling may also depend on the ratio of ERa /ERb (12).

Upon ligand activation, ERs induce genomic and non-genomic effects (13, 14). Non-genomic effects can be mediated through the G-protein-coupled estrogen receptor (GPER), also expressed in granulosa cells and involved in E2 induced VEGF expression (13). The genomic effects result in the regulation of gene transcription and occur through direct binding of ERs to estrogen responsive elements (EREs) in the regulatory regions of E2 target genes. Alternatively, ERs can interact with other transcription factors such as activating protein-1 (AP1) and stimulating protein-1 (SP1) to influence gene expression indirectly (10, 14, 15). Transcription factor binding sites (TFBS) for ERa and ERb have been mapped in MCF7 breast cancer cells through ChIP-PET and ChIP-on-chip analysis (11, 16), which identified 1,234 and 1,457 high confidence ER binding sites, respectively. Around 75% of all ER binding sites can be target by both ERa and ERb receptors. Interestingly, only 5% of ERb binding sites contains exclusively an ERE, but 60% of them contains AP-1 like binding sites combined with ERE-like sites, and 45% among these contains additionally forkhead family binding sites (11). The ratio between ERa and ERb is also able to change the capacity of ERb to bind its specific TFBS (17). Additionally, TFBS can be activated by ERa and ERb independent of ligand (i.e., 17b-estradiol) (18).

E2 can trigger apoptosis in human granulosa cells *via* binding to ERb1 (the only splice variant of human ERb able to bind the hormone) (19), but depending on the cell line, for example in human breast cancer cells, apoptosis can be triggered by E2 binding to ERa (20). Although apoptosis signals are not strong enough to justify regression of a cyclic canine CL (21), E2 has been implicated in human CL regression *via* apoptosis (22). Moreover, a recent study comparing regressing canine CL and pre-partum luteolysis indicated activation of estrogen receptors as one of the main represented functional terms related to structural changes in the regressing CL (23).

Collectively, canine CL expresses both *ESR1* and *ESR2*, and the ratio of *ESR1* /*ESR2* varies throughout diestrus. There is a greater *ESR1* expression in early-luteal phase (1) and greater *ESR2* expression in the late-luteal phase (6). However, the role of E2 and its receptors in regulating canine CL function is still unknown. We hypothesize that E2 binding to ERa and ERb is time-dependent and might activate different E2-responsive genes, and therefore, different biological functions throughout canine CL lifespan. The aim of the present study was (1) to access differentially expressed genes (DEG) in canine CL with over-represented transcription factor binding sites (TFBS) related to *ESR1* and *ESR2* to gather an idea of the presupposed broad action of E2 along diestrus, and (2) characterize luteal cell responses to ERa and ERb inhibition to gain further insights into the role of E2 in specific aspects of the canine CL physiology, particularly on its regression.

## Materials and Methods

### Animals and experimental design

Thirty healthy mongrel bitches were included in this study after approval by the Committee of Ethics in the Use of Animals of the School of Veterinary Medicine and Animal Science of the University of São Paulo (protocol number 2719/2012). After the onset of proestrus bleeding, blood samples were collected on alternate days to determine plasma progesterone (P4) concentrations. An additional blood collection for plasma P4 measurement was made on the day of surgery, prior to anesthesia. Ovulation was considered to have occurred when P4 plasma concentrations reached at least 5 ng/ml (24). The CLs were collected *via* ovariosalpingohysterectomy on days 10, 20, 30, 40, 50, and 60 post-ovulation (p.o.; *n* = 5 animals per day). After collection, CLs were dissected from the surrounding ovarian tissue and 3 CLs of each animal were frozen immediately in liquid nitrogen for total RNA extraction, qPCR and western blotting analysis; remaining CLs were fixed in 4% buffered formalin for 24 h and used for immunohistochemistry. For cell culture, all CLs collected on days 20, 40 and 60 p.o., from 12 different dogs were immediately washed and kept in sterile phosphate buffered solution prior to processing as described below.

### Hormone assay

Plasma progesterone concentrations were measured to define the day of ovulation using a validated chemiluminescence immune assay (Elecsys Progesterone III, Roche Diagnostics). The analytical sensitivity of the P4 assays was 0.10 ng/mL. The inter-assay coefficient of variation (CV) was 7.51% and the intra-assay CV was 6.11% as described by (25). The reagents used for P4 determinations came in the cobas^®^ e pack PROG3 (Roche Diagnostics).

### RNA-seq data analysis

RNA-seq data was generated and firstly analyzed as described previously (26), using CL of three different dogs per group. Data are publicly available at NCBI Gene Expression Omnibus under the number GSE89293. A total of 3,300 DEGs resulting from the contrasts 20 × 10, 30 × 10, 30 × 20, 40 × 10, 40 × 20, 40 × 30, 50 × 10, 50 × 20, 50 × 30, 50 × 40, 60 × 10, 60 × 20, 60 × 30, 60 × 40 and 60 × 50 were converted into their human orthologs using the Mammalian Annotation Database (MAdb: http://madb.ethz.ch), which is a collection of pairwise ortholog groups among human, cow, pig, horse, rabbit, mouse and dog genomes. Finally, we used oPOSSUM3 (27, 28) to identify the overrepresented, conserved TFBS related to ESRs. The same list containing 3,300 DEGs was submitted to oPOSSUM3 twice: in the first run, genes containing predicted TFBS for ERa/*ESR1* were shown, and in the second run, the ones containing predicted TFBS for ERb/*ESR2*. A gene was included in the DEG list if the false discovery rate (FDR) was < 0.01 and the respective *p*-value < 0.001. Ingenuity Pathway Analyses (IPA, Qiagen, Redwood City, CA, USA), revealed canonical pathways and upstream regulators for DEGs showing TFBS for ERa and ERb. A Venn Diagram was generated (https://bioinfogp.cnb.csic.es/tools/venny/index.html) to visualize upstream regulators related to both ESR1 and ESR2 (intersection).

### Quantitative real-time reverse transcription PCR

Total RNA was isolated from CL in different stages of diestrus by Trizol^®^ reagent (Life Technologies, Grand Island, NY, USA) according to manufacturer's instructions. Unless otherwise stated, all reagents and equipment were from Life Technologies. Concentration and quality of RNA were determined using a BioPhotometer (Eppendorf, Hamburg, Germany), and integrity was analyzed by electrophoresis through a 2% agarose gel. Following DNase treatment, 1 μg of total RNA (extracted from CLs) and 0.5 μg of total RNA per sample (extracted from luteal cells in culture) was reverse transcribed using Superscript III reverse transcriptase according to the manufacturer's instructions. DEPC-treated water was used as negative control. PCR reactions were performed with an automated fluorometer (ABI Prism^®^ 7500), using 96-well optical plates. Each sample (25 ng of total RNA) was analyzed at least in duplicate. Negative controls were set up by replacing cDNA with water. Validated genes were selected according to biological processes they participate in (cell proliferation, luteal maintenance, cell death, luteal regression) and the presence of TFBS for ERa and ERb. The gene-specific primers used are listed in Table 1. After evaluation of three different reference genes, glyceraldehyde 3-phosphate dehydrogenase (*GAPDH*), cyclophilin A (*PPIA*) and ribosomal protein L32 (*RPL32*), we used the NormFinder software (29), which selected *GAPDH* as the best reference gene for our analyses. The relative expression of estrogen receptor 1 (*ESR1*), estrogen receptor 2 (*ESR2*) lymphoid enhancer-binding factor 1 (*LEF1*), catenin-beta 1 (*CTNNB1*), cyclin D1 (*CNND1*), marker of proliferation Ki-67 (*MKI67*), N-Myc downstream-regulated *gene 2 (NDRG2), ATPase Na*^+^*/K*^+^ *(ATP1A1)*, caspase 3 (*CASP3)*, caspase 8 *(CASP8)*, caspase 9 *(CASP9)*, cell surface death receptor *(FAS)*, and BCL2 associated X apoptosis regulator *(BAX)*, cytochrome P450 family 19 subfamily A member 1 *(CYP19A1)*, cytochrome P450 family 11 subfamily A member 1 *(CYP11A1)*, hydroxy-delta-5-steroid dehydrogenase, 3 beta- and steroid delta-isomerase 1*(HSD3B1)* and solute carrier family two member *4 (SLC2A4)* was calculated as described previously (30) followed by linear regression (LingRegPCR 7.0) fluorescent analysis (31).

**Table 1:** List of qRT-PCR primers.

**Gene**	**Primer**	**Sequence**	**Probe**	**GenBank N°**
*LEF1*		cf02686726_mh		FJ374770.1
*CTNNB1*	Forward Reverse	5′ ACTGAGCCTGCCATCTGTGC 3′ 5′ TCCATAGTGAAGGCGAACAGC 3′	TTCGTCATCTGACCAGCCGACACCA	F*J2*68743.1
*CCND1*		cf02626707-m1		AY620434.1
*NDRG2*		Cf02722935_m1		XM_858273.1
*ATP1A1*		Cf02627969_m1		L42173.1
*GAPDH*		ID cf04419463_gH		AB038240.1
*CASP8*		ID cf02627553_ml		DQ223013.1
*CASP9*		ID cf02627331_ml		DQ116956.1
*CASP3*		ID cf02622232_ml		AB085580.1
*FAS*		ID cf 02651136_ml		XM_543595.2
*BAX*		ID cf02622186_ml		AB080230.1
*MKI67*		ID cf0263588_gl		XM_533319.2
*CYP11A1*		ID cf02635588_gl		XM_533319.2
*HSD3B1*	Forward	5′ TCCCCAGTGTTTCTGATTC 3′		AY739720.1
	Reverse	5′ CACCAACAAATGCACGATTC 3′		
*SLC2A4*	Forward Reverse	5′ GCCTGCCAGAAAGAGTCTGAAG 3′ 5′ GCTTCCGCTTCTCCTCCTT 3′	CAGTCCCCAGATACAT	NM_001159327
*ESR1*	Forward	5′CCTGCAAAGCCTTCAAGAG 3′	TCAATGCTCCCCTGGATGG	AJ313195.1
	Reverse	5′ GGAAGCCGGACAGCTGTAC 3′		
*ESR2*	Forward	5′ CCTGCAAGGCCTTCTTCAAGA 3′	CATCCAAGGGAACATC	AJ313196.1
	Reverse	5′GGCTGGGCAGCTGTACTC 3′		
*CYP19A1*	Forward	5′ GTACCGGCCTGACCAGTT 3′	CATGCCAGAGCGCTTC	NM_001008715.1
	Reverse	5′ ACTTAATGATGGAGAAGATGAGCTGACT 3′		

### Immunohistochemistry

Luteal tissue protein distribution of caspase 3, caspase 8, caspase 9 and BAX proteins, all involved in cellular death and most likely in canine luteal regression, was evaluated by an immunoperoxidase method on 2 μm tissue sections prepared from four CLs per dog, using one section per CL and four dogs per group to assure accuracy (32). The primary antibodies used were polyclonal anti-rabbit for caspase 3, caspase 8, caspase 9 and BAX (Table 2). Negative controls were prepared using rabbit IgG (Santa Cruz Biotechnologies, Dallas, TX, USA). Positive controls were mouse lymph node sections prepared according to the manufacturer's protocol [as previously shown by (33)].

**Table 2:** List of antibodies for immunohistochemistry and western blotting (WB).

**Antibody**	**Isotype**	**Immunogen**	**Dilution WB**	**Catalog no**
Caspase 3	Polyclonal rabbit IgG	Recombinant catalytically active human caspase-3	1:2500	IMGENEX (IMG-5700)
Caspase 8	Polyclonal rabbit IgG	Recombinant catalytically active human caspase-8	1:2500	IMGENEX (IMG-5703)
Caspase 9	Polyclonal rabbit IgG	Recombinant catalytically active human caspase-9	1:2500	IMGENEX (IMG-5705)
BAX	Polyclonal rabbit IgG	Full length recombinant mouse Bax	1:2500	IMGENEX (IMG-5684)

### Western blotting

CL samples were homogenized in buffer containing 50 mM potassium phosphate (pH 7.0), 0.3 M sucrose, 0.5 mM dithiothreitol (DTT), 1 mM ethylenediaminetetraacetic acid (EDTA, pH 8.0), 0.3 mM phenylmethylsulfonyl fluoride (PMSF), 10 mM NaF, and phosphatase inhibitor cocktail (1:100; Sigma-Aldrich). Total protein content was determined spectrophotometrically using the Bradford method (34), and calculated by interpolation of a standard curve constructed with increasing concentrations of albumin, read at 595 nm. For each sample, 50 micrograms of total protein were resolved on 15% SDS–PAGE minigels and electrophoretically transferred onto polyvinylidene difluoride membranes (PVDF, Bio-Rad Laboratories, Hercules, CA, USA). CASP3, CASP8, CASP9, and BAX were detected with specific antibodies (Table 2) and visualized using an Enhanced Chemiluminescence (ECL) kit (Amersham Biosciences, Piscataway, NJ, USA). Images were captured by ChemiDoc MP Image system (Bio-Rad Laboratories) and normalized to the abundance of actin-beta (ACTB; 42 kDa) using ImageJ Software (Bio-Rad Laboratories).

### Cell culture

Cell culture was performed to verify the effects of ERa and ERb blockers on canine luteal cells derived from different timepoints in diestrus. Canine luteal cells were isolated from twelve healthy mongrel female dogs at early (day 20 p.o.), mid (day 40 p.o.), and late diestrus (day 60 p.o.; *n* = 4 animals/group). After washing with fresh phosphate buffered saline (PBS) containing 1% antibiotic-antimycotic solution (A5955, Sigma-Aldrich), CLs were minced. Fragments were transferred to 1 ml Dulbecco's modified Eagle's medium (DMEM) supplemented with 5% fetal bovine serum (FBS; Sigma-Aldrich), 1% L-glutamine (Sigma-Aldrich.), 20 mM HEPES (Sigma-Aldrich), 1% antibiotic-antimycotic solution (A5955, Sigma-Aldrich), and 1 mg/ml collagenase type 1 (C0130; Sigma-Aldrich). Samples were incubated for 1 h with shaking (60 shakes/min) at 37°C. The suspension was centrifuged at 200 × g for 10 min, re-suspended in DMEM, and filtered through a cell strainer (70 μm; BD Falcon; BD Biosciences, Durham, NC, USA). The filtrate was centrifuged at 200×g for 10 min, re-suspended in DMEM (v/v) for 10 min, centrifuged at 200 × g for 10 min, and re-suspended in DMEM. Subsequently, cells were seeded in 24-well plates and incubated (5% CO2) at 37°C until 90% confluence.

### 17b-estradiol, MPP and PHTPP treatment

After cultures reached 90% confluence, cells were serum-starved for 24 h. Cultures were divided into six groups: Control, E2 (treated with 100 nM 17β-Estradiol; (Sigma-Aldrich; E2), ERa block (treated with 10 nM methyl-piperidone-pyrazole; TOCRIS Biosciences, Bristol, UK;), ERb block (treated with 10 μM pyrazole (1,5-a) pyrimidine; TOCRIS Biosciences; PHTPP), E2 + ERa block (treated with E2 + MPP), and E2 + ERb block (treated with E2 + PHTPP). To determine which concentration of MPP and PHTPP should be used, dose-response curves were performed and minimal concentrations necessary to achieve stimulation of CYP19A1 expression were chosen. For RNA preparations, culture medium was discarded and 1 ml of TRIzol^®^ was added to the cells, followed by scraping of the cell layer, freezing in liquid nitrogen, and storage at −80°C until further processing by qRT-PCR.

### Statistical analysis

Data were tested for homogeneity of variance and normality of residues using the F-test and the Kolmogorov-Smirnov test, respectively. Data are presented as mean ± SEM. The qPCR and hormone data were compared by one-way analysis of variance (ANOVA), for the main effect of day, followed by the Bonferroni correction for normally distributed data. Differences were considered statistically significant when the *p*-value was < 0.05. All statistical analyses of validation procedures were performed using GraphPad Prism 5 (GraphPad Software, Inc., San Diego, CA, USA).

## Results

### *ESR1* and *ESR2* gene expression in the canine CL during diestrus

**Figure 1:**
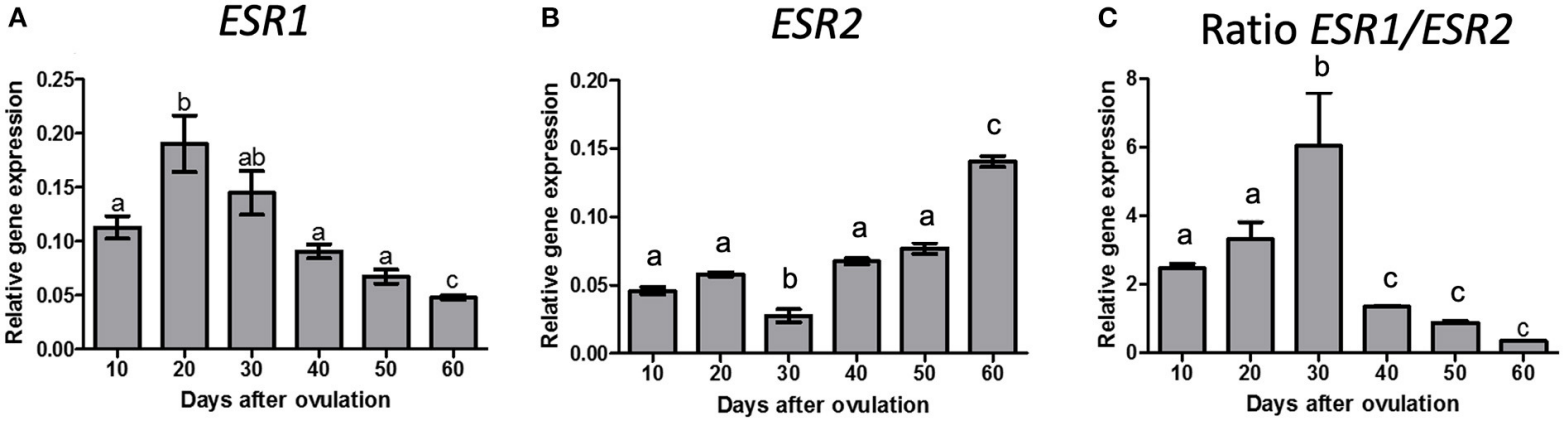
Gene expression of ESR1 **(A)**, ESR2 **(B)** and ratio of ESR1/ESR2 **(C)** in canine CL during diestrus (10 to 60 days p.o.). Data are presented as mean ± standard error of relative gene expression (*n* = 4 animals/group). Bars with different letters indicate significant differences among groups (*P* < 0.05).

The *ESR1* (Figure 1A) and *ESR2* (Figure 1B) mRNA expression changed significantly during diestrus (*P* < 0.0001); *ESR1* expression was greater on day 20 than on day 10 p.o. (*P* < 0.0001) decreasing on day 40 and further on day 60 p.o., whereas *ESR2* decreased from day 20 to 30 p.o., increased from day 30 to 40 p.o. and increased further on day 60 p.o. *ESR1*/*ESR2* ratio (Figure 1C) shows an increased from day 20 to 30 p.o. and a decrease from day 30 to 40 p.o., remaining low until day 60 p.o.

### Transcription factor binding sites related to the E2 receptors

In a previous work of our group, we compared the temporal gene expression among days 10, 20, 30, 40, 50 and 60 p.o. The analysis revealed the presence of 3300 DEGs in at least one comparison (26). We converted these DEGs into their human orthologs in order to identify the over-represented TFBS related to ERs. In our ortholog approach for the transcription factor (TF) analysis, we assumed that the TF binding sites are evolutionarily conserved, as demonstrated previously (35). Seventy-seven DEGs exhibited TFBS for ERa and 450 exhibited TFBS for ERb (Supplementary Tables 1, 2), whereas TFBS for both ERa and ERb were found in 67 DEGs.

Genes presenting TFBS for ERa were related to several canonical pathways (Supplementary Table 3), among which the most significant, based on -log *p*-value, were G beta gamma signaling, sulfite oxidation, glycine biosynthesis, retinol biosynthesis, insulin signaling pathway, growth hormone signaling. ERb TFBS were encountered in genes participating in canonical pathways related to epithelial adherens junction signaling, nitric oxide signaling, GABA receptor signaling, signaling by Rho family GTPases, among others (Supplementary Table 3). We found 384 and 778 upstream regulators for ERa and ERb TFBS, respectively, from which 204 were common to both receptors (Supplementary Table 3). Different miRNAs, E2, P4, interleukins, *PPARG, MAPK1*and *CTNNB1* were found among the upstream regulators of both ERa and ERb TFBS-containing genes. *CCND1* and *LEF1* were found among the upstream regulators for ERb and ERa TFBS-containing genes, respectively. Moreover, *LEF1, NDRG2* and *ATP1A1* participated as target molecules in several intracellular pathways triggered by ESR2 upstream regulators, including 17b-estradiol.

### Validation of mRNA levels of selected genes by qPCR

The selected genes included upstream regulators for *ESR1* (*LEF1*), *ESR2* (*CTNNB1* and *CCND1*), genes regulated by E2 (*NDRG2* and *ATP1A1*), proliferation markers (*MKI67*) and apoptosis markers (*CASP3, CASP8, CASP9, BAX* and *FAS*), which expression has not yet been shown in canine CL along diestrus. Additionally, we measured RNA expression of genes related to glucose uptake (*SLC2A4*) and steroidogenesis (*CYP19A1, CYP11A1* and *HSB3B1)* after E2 receptor inhibition in our cell culture model, from which the *in vivo* expression in canine CL was published elsewhere (1, 36, 37).

**Figure 2:**
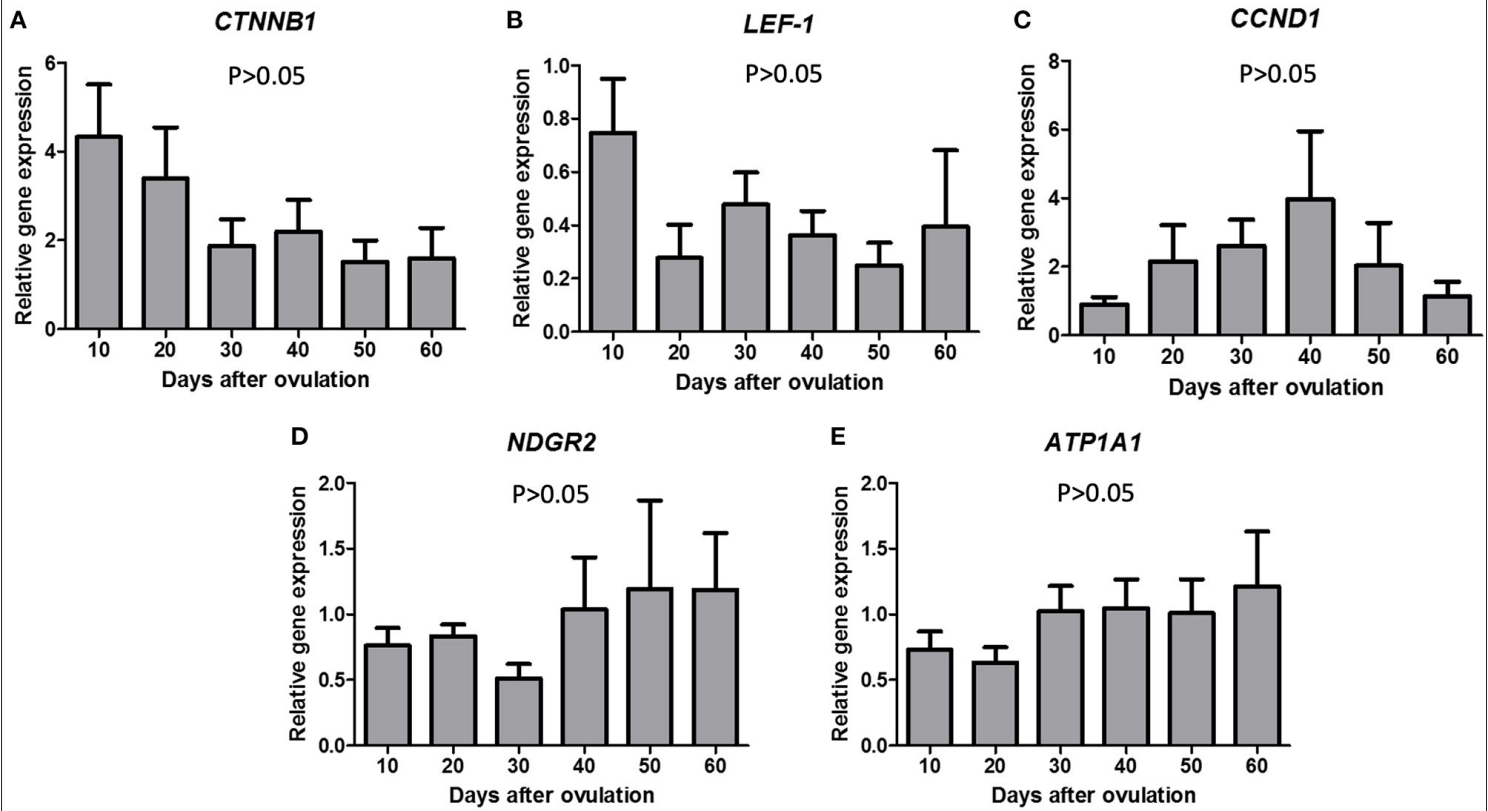
Gene expression of CTNNB1 **(A)**, LEF-1 **(B)**, CCND1 **(C)**, NDGR2 **(D)**, and ATP1A1 **(E)** in canine CL during diestrus (10 to 60 days p.o.). Data are presented as mean ± standard error of relative gene expression (*n* = 4 or 5 animals/group). No difference among groups were observed (*P* > 0.05).

No significant differences in mRNA expression were observed for *LEF1, CTNNB1, CCND1* (Figures 2A–C) or *NDGR2* and *ATP1A1* during diestrus (Figures 2D,E).

**Figure 3:**
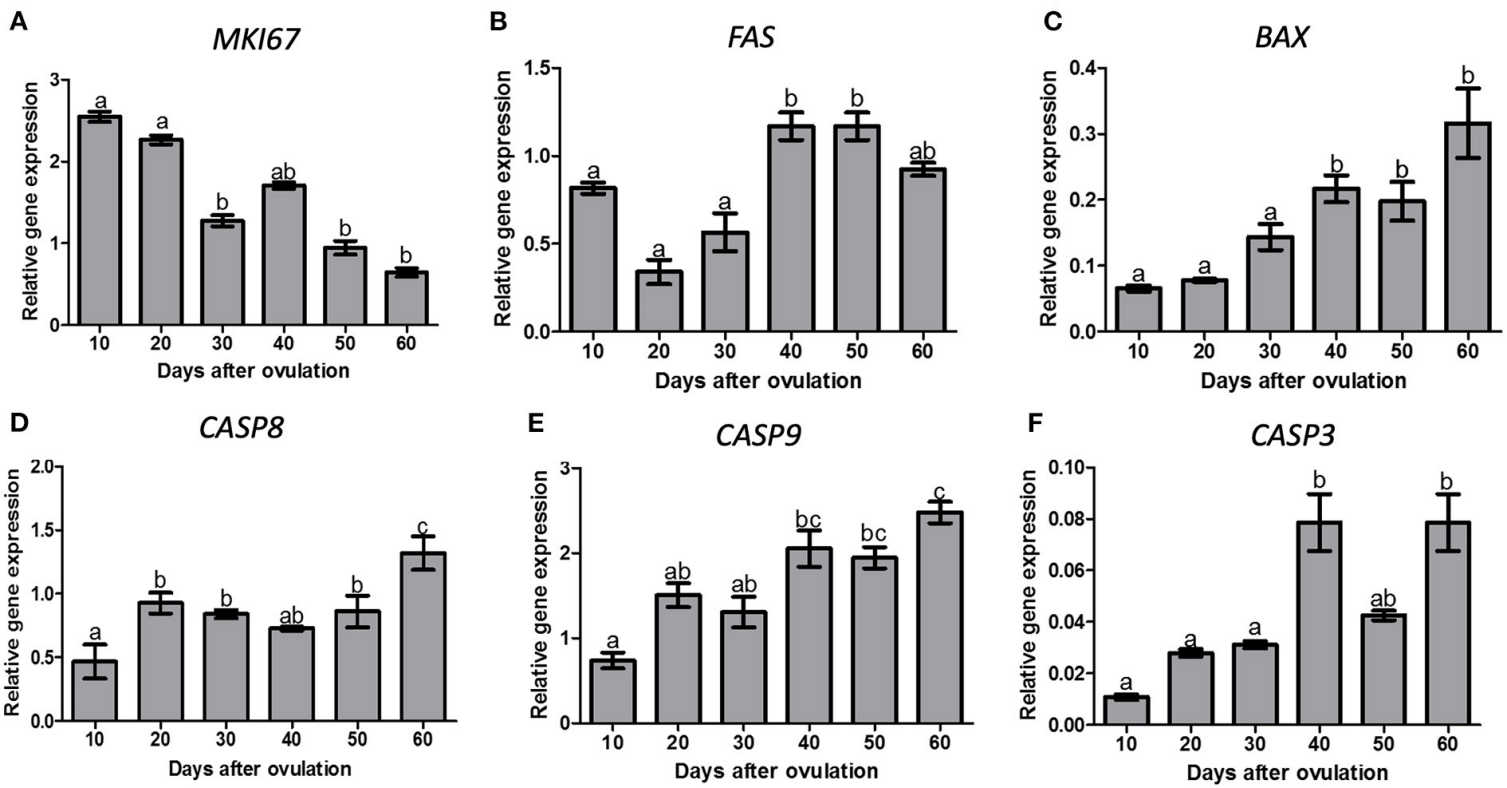
Gene expression of, MKI67 **(A)**, FAS **(B)**, BAX **(C)**, CASP8 **(D)**, CASP9 **(E)** and CASP3 **(F)** in canine CL during diestrus (10 to 60 days p.o.). Data are presented as mean ± standard error of relative gene expression (*n* = 4 or 5 animals/group). Bars with different letters indicate significant differences among groups (*P* < 0.05).

*MKI67* expression decreased from day 20 to 30 p.o. and remained lower until day 60 p.o. (*P* < 0.05; Figure 3A). *FAS* expression showed highest expression levels on days 40 and 50 p.o. (*P* = 0.0002, Figure 3B). *BAX* expression increased from day 30 to day 40 p.o. (*P* < 0.0001), being greater on the second half of diestrus (Figure 3C).

There was an effect of time (*P* < 0.0001) on *CASP8*, which increased on day 20 and further on day 60 p.o. (Figure 3D). *CASP9* expression increased on day 40 and 60 p.o. in comparison to day 10 p.o. (*P* < 0.0001), reaching maximum values on day 60 p.o. (Figure 3E). *CASP3* expression increased from day 30 to 40 p.o., reaching maximum values on days 40 and 60 p.o. (*P* < 0.05, Figure 3F).

### Caspase 3, caspase 8, caspase 9 and BAX protein localization in the canine CL during diestrus

**Figure 4:**
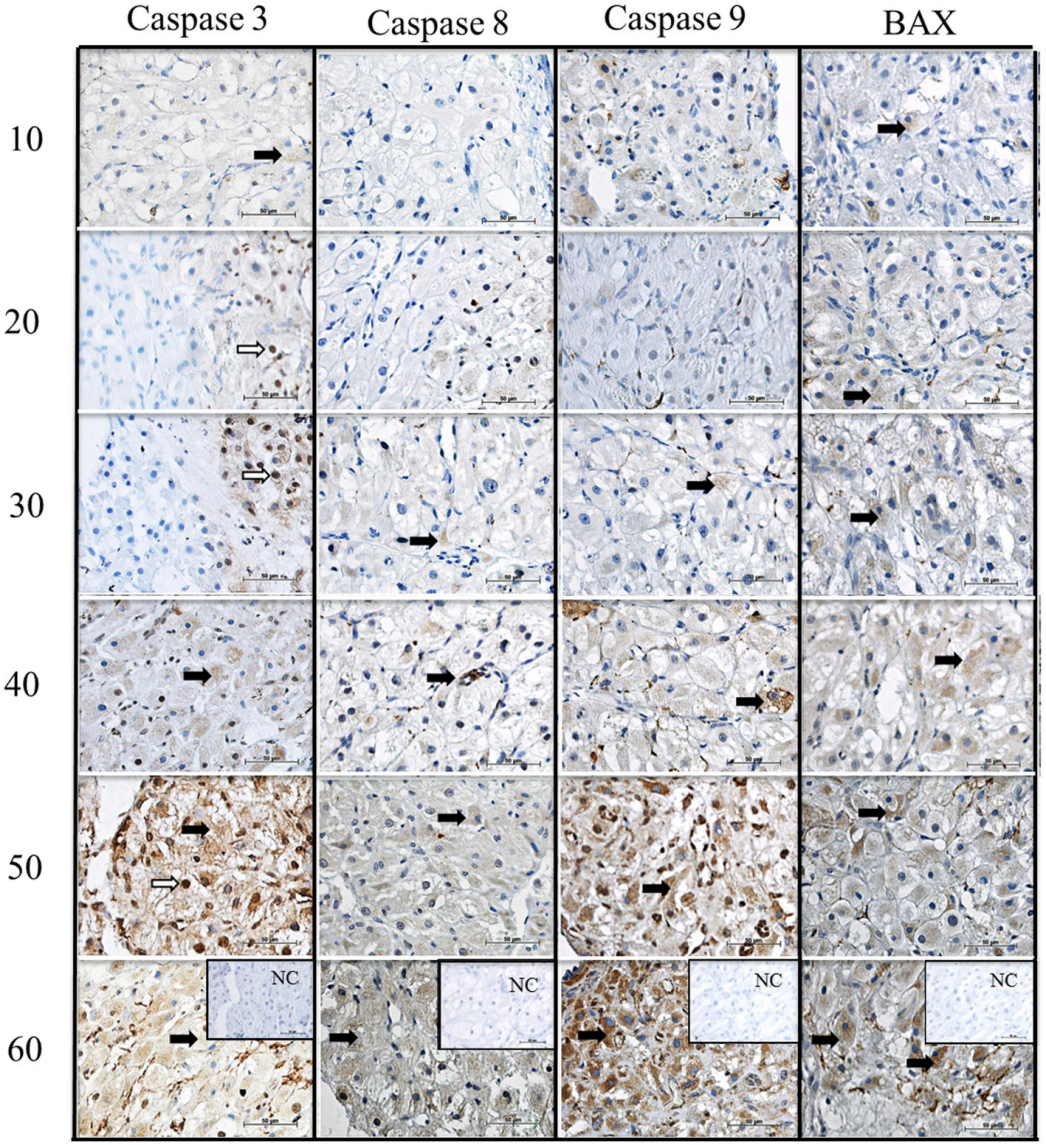
Immunolocalization of CASPASE 3, CASPASE 8, CASPASE 9 and BAX in canine CL during diestrus. Lines 10, 20, 30, 40, 50, 60, days after ovulation. NC, negative control. Black arrows indicate the cytoplasmic and white arrows indicate nuclear staining. Scale bar 50 μm.

We verified the localization of apoptosis-related proteins (caspase 3, caspase 8, caspase 9, and BAX) in the canine corpus luteum over diestrus. Caspase 3 staining could be observed in the cytoplasm and nucleus of luteal, endothelial, and stromal cells from day 10 to day 60 p.o. (Figure 4). Caspase 8, caspase 9, and BAX followed the same expression pattern as caspase 3; however, the nuclear staining was not evident. Although immunohistochemistry is not a quantitative method, and despite the background staining observed, in particular at later luteal stages, the intensity of signals appeared to increase over time, matching the western blotting results described below.

### Caspase 3, caspase 8, caspase 9 and BAX protein expression in the canine CL during diestrus

**Figure 5:**
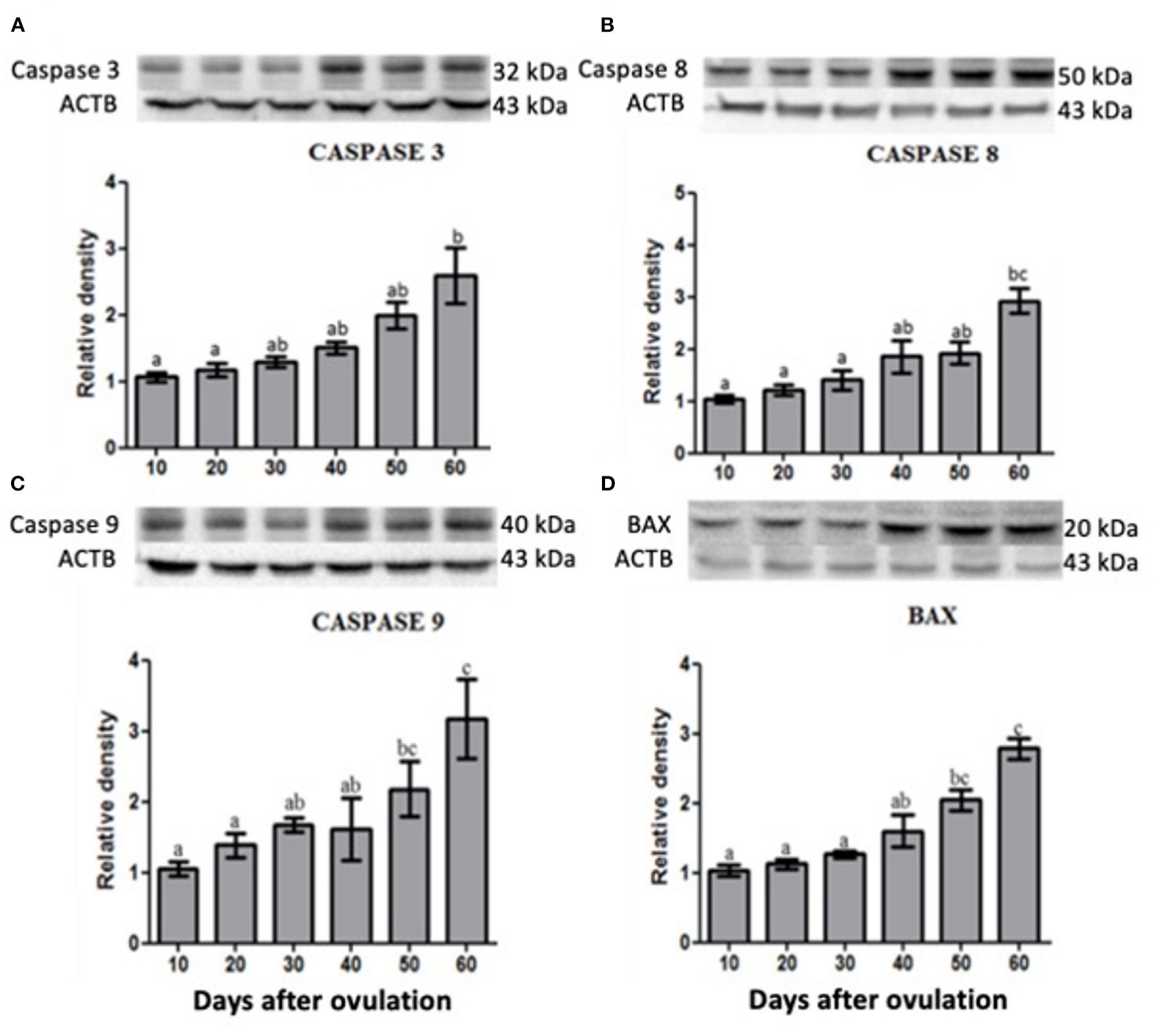
Protein expression of Caspase 3 **(A)**, Caspase 8 **(B)**, Caspase 9 **(C)** and BAX **(D)** in canine corpus luteum. Representative blots on top of each column, which represent mean ± standard error (*n* = 4/group). Different letters indicate significant differences among groups (*P* ≤ 0.05).

Western blotting analysis revealed that caspase 3, 8 and 9 as well as BAX expression was increased at the end of diestrus and the highest or greater expression was observed on day 60 p.o. (Figure 5).

### *CASP3, CASP8, CASP9, BAX, FAS, MKI67, CYP19A1, CYP11A1, HSB3B1*, and *SLC2A4* gene expression in the canine luteal cells in culture after inhibiting ERa and ERb

**Figure 6:**
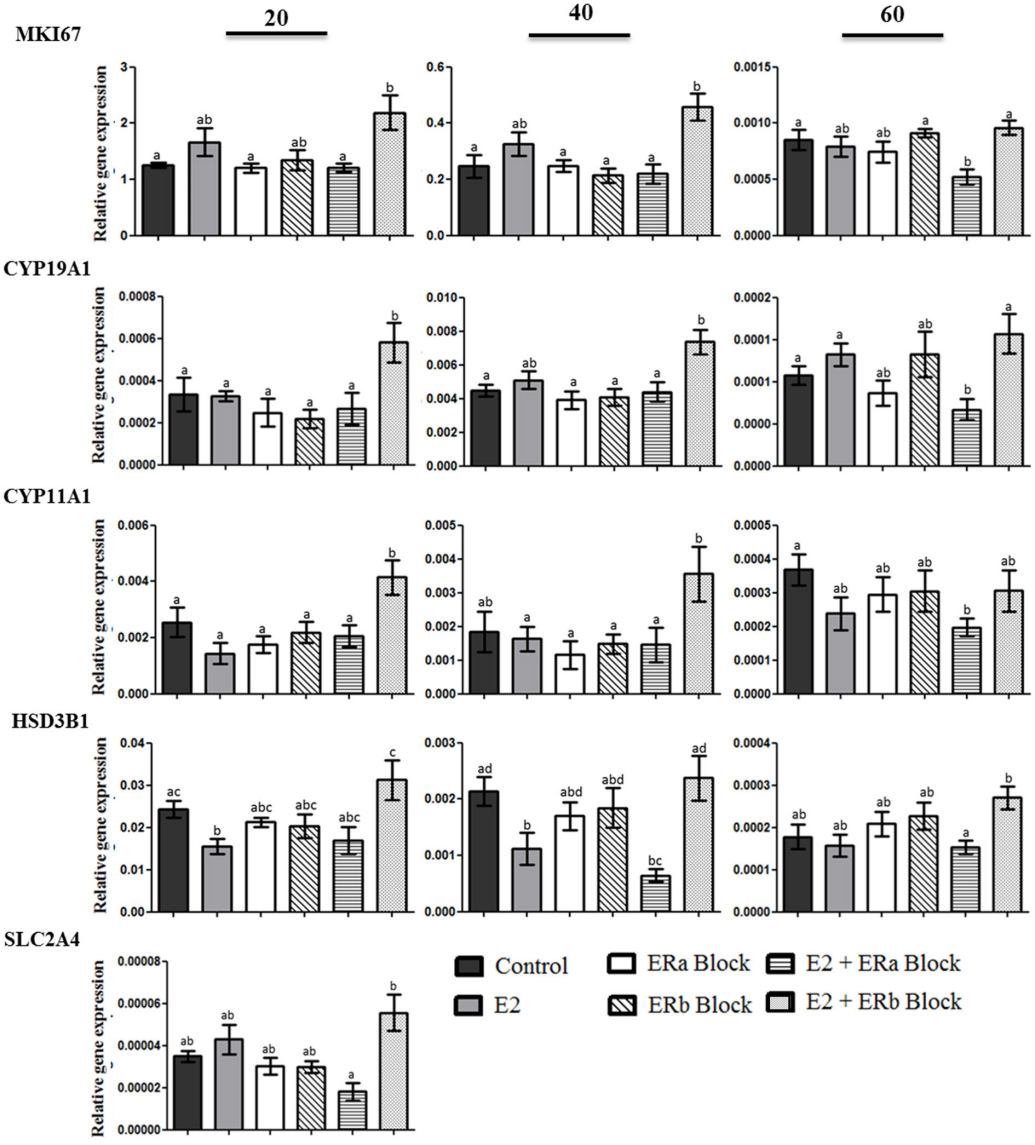
Gene expression *MKI67*, cytochrome P450, family 19, subfamily A, polypeptide 1 (*CYP19A1*), cytochrome P450, family 11, subfamily A, polypeptide 1 (*CYP11A1*), 3-β-hydroxysteroid dehydrogenase/Δ-5-4 (*HSD3B1*), solute carrier family 2 (facilitated glucose transporter), and member 4 (*SLC2A4*) in luteal cells collected at 20, 40, and 60 days after ovulation in diestrous bitches. Bars indicate six different groups: Control (no treatment), E2 (treated with 100 nM E2), ERa block (treated with 10 nM methyl-piperidone-pyrazole [MPP]), ERb block (treated with 10 uM (1,5-a) pyrimidine [PHTPP]), E2 + ERa block (treated with E2 + MPP), and E2 + ERb block (treated with E2 + PHTPP). Data represent mean ± standard error of relative gene expression (*n* = 4 animals/group). Bars with different letters indicate significant differences among groups (*P* < 0.05).

We studied genes associated with the specific inhibiting of E2 receptors in luteal cells in three different stages of diestrus: full secretory activity (day 20), early and late luteal regression (days 40 and 60 p.o., respectively). The mRNA expression of genes related to proliferation (*MKI67*), steroidogenesis (*CYP11A1, CYP19A1* and *HSD3B1*) and glucose uptake (*SLC2A4*) was evaluated after inhibiting ERa and/or ERb (Figure 6). *MKI67* gene expression was increased when canine luteal cells were treated with 17b-estradiol plus ERb inhibiting; however, it was decreased under 17b-estradiol plus ERa inhibition. This suggests that E2 effects on *MKI67* happened through the ERa receptor. *CYP19A1* and *CYP11A1* followed the same pattern of *MKI67* response to ERa and ERb inhibition.

There was a significant decrease in the expression of *HSD3B1* when canine luteal cells collected on days 20 and 40 were treated with E2, whereas luteal cells treated with E2 plus ERb inhibition showed a significant increase in the relative expression of *HSD3B1*. The expression of *SLC2A4* was identified only in cells collected on day 20 p.o. The glucose transporter 4 transcript was up-regulated in cells treated with E2 + ERb inhibition compared to the E2 + ERa inhibition, emphasizing again the luteotropic effects of ERa.

**Figure 7:**
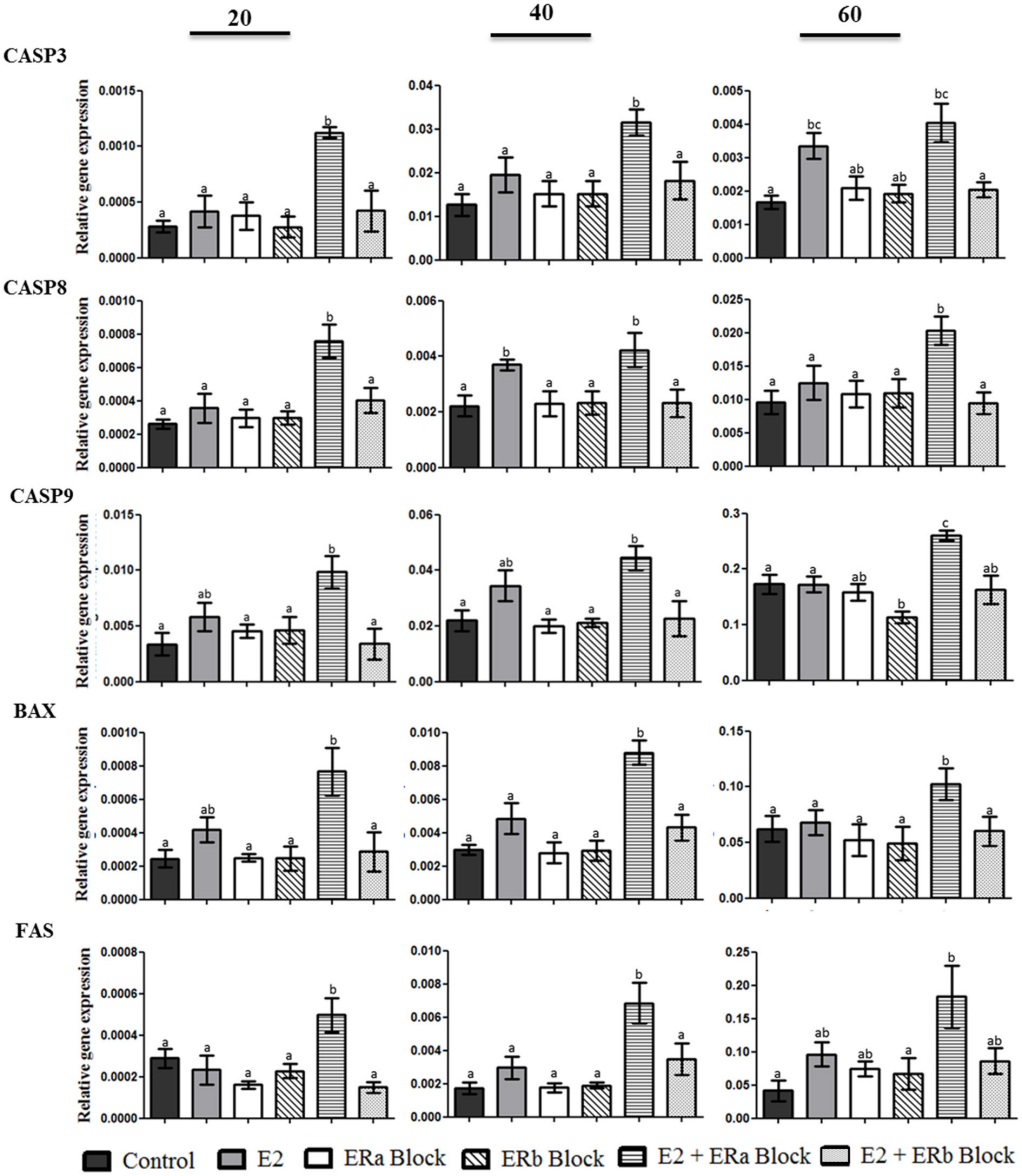
Gene expression of *CASP3, CASP8, CASP9, BAX*, and *FAS* in canine luteal cells collected on days 20, 40, and 60 after ovulation and cultivated for 10 days until confluence was reached. Bars indicate six different groups: Control, E2 (treated with E2), ERa block (treated with methyl-piperidino-pyrazole [MPP]), ERb block (treated with pyrazolo (1,5-a) pyrimidine [PHTPP]), E2 + ERa block (treated with E2 + MPP), and E2 + ERb block (treated with E2 + PHTPP). Data represent mean ± standard error of relative gene expression (*n* = 4 animals/group). Bars with different letters indicate significant differences among groups (*P* < 0.05).

Expression of apoptosis associated genes in luteal cells after treatment is shown in Figure 7. *CASP3* gene expression was increased when canine luteal cells were treated with 17b-estradiol plus ERa block. However, luteal cells collected on day 60 p.o. also showed an increase in CASP3 when treated with E2 alone if compared to control cells. *CASP8, CASP9, BAX*, and *FAS* expression followed the same pattern of response to ERa and ERb inhibition, i.e., they always increase under E2 + ERa inhibition but not under ERa inhibition without E2 treatment; moreover, *CASP8* showed a significant increase on day 40 under the stimulus of E2 alone, which was not observed for the other apoptosis-related genes in any studied phase.

## Discussion

Due to its long lifespan, as well as some uncertainties regarding the role of 17b- estradiol in its control, the canine CL has been chosen to study diestrus-related 17b-estradiol actions. The present study was based in the concept raised by Papa and Hoffmann (1) that the CL is both source and target of steroid hormones. Our transcriptome results followed by oPOSSUM analysis, revealed predicted DEGs over diestrus with enriched TFBS for the E2-receptor complex, suggesting E2 is involved both in the proliferative and regression phases of the canine CL. Although there are limitations of this analysis based on human ortholog genes, the assumption of evolutionarily conserved TFBS seems to be plausible (35). Such contrasting roles were possibly mediated by the selective binding of E2 to ERa and ERb, as well to the switch on *ESR1*/*ESR2* ratio observed from day 30 to 40 p.o. Moreover, the inhibition of either ERa or ERb in canine luteal cells added to our understanding of the possible roles of both receptors, as described previously for other species (38) and tissues (39, 40). Comparative aspects of E2 on CL function in dogs and other species have also been recently reviewed in (41).

Increased expression of *ESR1* and consequently of the *ESR1*/*ESR2* ratio has been shown to be associated to aggressive prognostic and worse overall survival in patients with papillary thyroid carcinoma (42), whereas decreased *ESR1*/*ESR2* ratio in endometriosis-like phenotype mice lead to low response to P4 and subfertility (43). Moreover, during physiological development of rat Sertoli cells, it has been reported that *ESR1*/*ESR2* ratio decreases with age and this shift seems to be important for termination of proliferation and begin of differentiation period (44). Also in canine CL the ratio *ESR2*/*ESR1* has been shown to be dependent on the pregnancy and developmental stages (6) (also addressed below). These previous studies, using different species, cells, and organ models, further highlight the different biological effects mediated by the two E2 receptors. Similarly, our results show a switch of *ESR1*/*ESR2* ratio from day 30 to 40 p.o. matching the end of CL maintenance phase and the start of the regression phase. Sonnack (45) used transmission electron microscopy to illustrate morphological changes in canine non-pregnant CL on day 45 p.o. (early luteal regression): the smooth endoplasmatic reticulum of luteal cells shows the first morphological changes and signs of degenerative transformation accompanied by fatty degeneration, mirrored by the deposition of lipid vacuoles in the cytoplasm.

Cancer related research corroborates the idea of ERa acting as a proliferative mediator and ERb as an anti-proliferative agent (42). Moreover, when present in the same cell, as in the case of canine luteal cells, ERb induces the formation of ERa/ERb heterodimers that are less active than ERa homodimers, and thereby work as an ERa repressor (46). Recently, the *ESR2*/*ESR1* ratio was reported for the gestational and non-gestational canine CL (6) and, although not matching completely the ratio reported in the present work, one could observe the mentioned transition in the abundance of transcripts reflected in the increased *ESR2/ESR1* ratio (6). More in detail, authors describe a gradual and significant increase in the *ESR2/ESR1* ratio between days 10 and 30, and further toward day 40 p.o. followed by a decrease toward the end of luteal phase (6). Interestingly, in the same study (6), the abundance of *SULT1E1* was assessed and was increased during late dioestrus, at days 50 and 60. Based on that, the authors proposed the functional involvement of *SULT1E1* (converts active oestrogens into biologically inactive estrogen sulfates) in the functional withdrawal of E2 in regressing CL (6). Similar conclusions were implied from transcriptomic studies, showing increased expression of *SULT1E1* in regressing CL (23).

Our results from canine luteal cells in culture demonstrated that E2 + ERb block can induce an increase in *MIK67* mRNA expression on days 20 and 40 p.o. in comparison to control group, which cannot be achieved by E2 alone or in combination with ERa block (Figure 6, *p* < 0.05). The same pattern was observed for mRNA expression of steroidogenic enzymes (*CYP19A1* and *CYP11A1*). These mRNAs encode luteotropic proteins (1, 36, 37) aimed to drive luteal function, i.e., progesterone production to its plenitude. Besides LH and prolactin, insulin can also be considered an endocrine luteotropic factor in the canine CL (36) and its contribution to E2 production cannot be ruled out (25). The mechanism of E2 binding to ERa and leading to proliferation is opposed to binding to ERb (47) leading to expression of anti-proliferative genes such as *p-53, PI3K* and *Akt*, as well as the increase of stress associated and apoptosis related proteins (48), as also observed in our study.

Although caspase expression is normally associated with apoptosis, other functions in homeostasis have been attributed to them: e.g., caspase 3 participates in bone marrow stromal stem cell differentiation together with caspase 8, which is also involved in T-cell maintenance (49), both mechanisms dissociated from apoptosis. Caspases also participate as regulators of tumorigenesis, since genes involved in cell death have normally a tumor suppressor function (49). This explanation sounds reasonable to justify the increasing amounts of caspases and BAX observed in the second half of diestrus, especially in the phase of late luteal regression (day 60 p.o.), emphasizing the role attributed to E2 binding to ERb, which leads to an anti-proliferative effect. It is worth mentioning that the canine non-pregnant CL does not show signs of apoptotic degeneration during regression (45) and no over-represented biological function was associated with apoptosis when analyzing transcriptomic data (23, 26). On the contrary, the canine pregnant CL shows apoptosis as one of the over-represented biological functions during luteolysis (23).

These findings correlate very nicely with *in vivo* E2 plasma concentration (5) and ERs expression, which acquired an opposite pattern after day 40 p.o. The authors hypothesize that a slight E2 increase as well as the switch of increasing to diminishing *ESR1*/*ESR2* ratio support the canine CL to initiate programmed regression mechanisms, which deserves further experimental confirmation, especially because E2 concentrations used in cell culture, which were able to elicit a response, are above the plasma physiological concentrations. In the absence of E2-ERb drive, the canine diestrous CL could deviate from the physiologic path and go into uncontrolled proliferative conditions, as described for some breast cancers in which ERb expression was dysregulated (50).

In general, inhibition of ERa or ERb in the absence of E2 did not affect gene expression. In contrast, under E2 influence, ERa inhibition permitted upregulation of anti-proliferative factors such as caspases, *BAX* and *FAS*, and ERb inhibition stimulated transcription of proliferative and luteotropic factors such as *MIK67* and steroidogenic enzymes, suggesting the need of the ligand to promote different biological functions. Based on the presented results, it could be hypothesized that manipulating the functionality of ERs could provide a good future tool to regulate luteal life span in dogs, which certainly deserves further research.

No significant difference was observed for mRNA expression validated by qPCR of the selected upstream regulators (*LEF1, CTNNB1* and *CCND1*) of ERa and ERb, or E2 target molecules (*NDRG2* and *ATP1A1*). RNAseq analyses were carried out with 3 samples per group, and when adding another three samples for the validation process, no significant difference was observed. Other studies have reported a high variation in gene and protein expression among canine CL samples from the same stage of diestrus (26, 51) not always matching RNAseq data, which made the present observations not surprisingly. Nevertheless, in accordance with the bioinformatic analyses performed for the above-mentioned genes, which were differentially expressed over canine diestrus, as depicted from RNAseq, and presented a TFBS for ERa and/or ERb concomitantly, it was possible to visualize canonical pathways (Supplementary Table 3) in which *LEF1, CTNNB1, CCND1, NDRG2* and *ATP1A1* might be part during canine CL lifespan.

As target molecule for E2, *NDGR2* was qualitatively (qPCR) and quantitatively (RNAseq) more expressed after day 40 p.o. in canine CL. It is considered a tumor suppressor gene, activated in non-cancer cells under stress situations, leading to suppression of cell proliferation, protein synthesis and inducing cell death (52). Besides being a target molecule for E2, *via* binding to ERb, which in turn regulates *NDGR2* expression *via* transcriptional activation (53), it participates in other canonical and non-canonical pathways, such as *CTNNB1* and *NF-kb*, respectively; both genes also present a TFBS for *ESR2*, which expression in this and other study (6) increases after day 40 p.o. In many malignant tumors, *NGDR2* is able to suppress endothelial cell proliferation and enhance apoptosis by increasing p53 expression (54, 55). The highest p53 gene expression in canine non-pregnant CL was found on day 60 p.o. (26), suggesting a possible contribution of E2 in CL regression mediated by ERb, involving also the apoptotic mechanism and corroborating our functional studies in canine luteal cell culture.

In the present study *ATP1A1* expression increased qualitatively just before the structural regression started and it has already been reported to show a positive correlation with *NDGR2* protein and gene expression (56). In porcine preovulatory luteinized follicles, *ATP1A1* was functionally classified as a cell growth inhibitor (57). A decrease in *ATP1A1* expression has also been observed in several human cancers such as prostate, kidney and bladder, which lead to accelerated proliferation (56, 58, 59). The increased expression of *ATP1A1*, as seen in our RNAseq data, points toward an activation of *ATP1A1* transcription after binding of E2 to ERb, which seems to be necessary to reduce canine CL proliferation and initiate regression.

The *CTNNB1* gene, encoding for catenin b, and *CCND1*, encoding for cyclin D1, are in one hand direct upstream regulators of *ESR1* and *ESR2*. According to their pattern of expression depicted by RNAseq and their function (Supplementary Table 3), they act as repressors of *ESR2* and enhancers of *ESR1* transcription in canine CL in the first half of diestrus. It was also reported that *LEF1* can act as transcriptional repressor for E2 by competing with ERs to bind to DNA (60). LEF1 also participates in several canonical pathways driven by *ESR2* upstream regulators (Supplementary Table 3). Although its mRNA expression did not show time-dependent differences, RNAseq data pointed toward an increased expression in the beginning of diestrus, which corroborates its presumable repressive action on *ESR2*. On the other hand, E2 and related compounds have been shown to exert their proliferative effects by binding to ERa (61), inducing for example *CCND1* transcription, a key regulator of cell cycle progression (62–64). Cell proliferation was reported to be greater in the first half of diestrus and highest until day 15 p.o. in canine non-pregnant CL (1), matching our reported *ESR1/ESR2* ratio, E2 plasma concentrations (5) and cell culture inhibiting assays (Figure 6). It is noteworthy that new approaches we used to unravel E2 mechanisms of action in the canine CL brought complementary data in complete agreement with previously published data.

In conclusion, E2 plays a pleiotropic role in canine corpus luteum, from formation until regression. Several genes and proteins are affected by E2 through its binding to ERs in a time-dependent manner. The number of predicted genes differentially regulated over diestrus in the canine CL showing transcription factor binding sites for *ESR1* and *ESR2* points toward a much broader role of E2. Additionally, the *ESR1/ESR2* ratio associated with E2 fluctuations (5) over diestrus suggests possible underlying regulatory mechanism involved in autocrine and paracrine regulation of canine CL lifespan.

## Data Availability Statement

The original contributions presented in the study are included in the article/Supplementary material, further inquiries can be directed to the corresponding author/s.

## Ethics Statement

The animal study was reviewed and approved by Committee of Ethics in the use of Animals of the School of Veterinary Medicine and Animal Science of the University of São Paulo (protocol number 2719/2012). Written informed consent was obtained from the owners for the participation of their animals in this study.

## Author contributions

AB: formal analysis, investigation, data curation, and writing-original draft. AC: formal analysis, sample collection, and data curation. RS and LS: formal analysis, investigation, and data curation. IG: sample collection, validation, investigation. MB and SB: investigation, review, and editing the manuscript. MK: review and editing the manuscript. PP: conceptualization, formal analysis, investigation, data curation, writing, funding acquisition, and supervision. All authors contributed to the article and approved the submitted version.

## Funding

This research was supported by FAPESP (Grant Numbers 2011/22173-9 and 2014/00739-9).

## Conflict of interest

The authors declare that the research was conducted in the absence of any commercial or financial relationships that could be construed as a potential conflict of interest.

## Publisher's note

All claims expressed in this article are solely those of the authors and do not necessarily represent those of their affiliated organizations, or those of the publisher, the editors and the reviewers. Any product that may be evaluated in this article, or claim that may be made by its manufacturer, is not guaranteed or endorsed by the publisher.
